# Ice as a Local Anæsthetic

**Published:** 1853-10

**Authors:** 


*Ice as a Local Anœsthetic.*—For some time past, the French surgeons have been using a mixture of pounded ice and salt to produce local anæsthesia. This has been employed especially in that extremely painful operation, the removal of the toe and finger-nails.

The ice is pounded finely and then mixed with a sufficient quantity of dry salt, enveloped in a cloth, (one of thin oiled silk we should think preferable,) and applied to the part. It should not remain longer than five or six minutes, and the operation should be commenced immediately upon its removal. The application, simple as it is, disarms the operation of all its terrors, the patient witnessing it with the same composure as the bystanders. No ill-effects have been known to follow its employment.

Could it not be made to supersede chloroform in some dental operations?

